# Rigorous review and editorial oversight of clinical preprints

**DOI:** 10.7554/eLife.67528

**Published:** 2021-06-16

**Authors:** Mone Zaidi, Diane M Harper, Anna Akhmanova, Detlef Weigel, Timothy E Behrens, Michael B Eisen

**Affiliations:** 1 eLifeCambridgeUnited Kingdom

**Keywords:** medicine, public health, physician-scientists, peer review, preprints

## Abstract

Research in many different areas of medicine will benefit from new approaches to peer review and publishing.

The urgent need to understand and control COVID-19, and to effectively treat people with the disease, has mobilized global scientific and medical communities like nothing in human history. It has also ushered in a new era in medical publishing. Notably, the desire to share results rapidly, widely and openly has led to an explosion of submissions to the preprint server medRxiv.

But even as the pandemic has demonstrated the potential for rapid, author-driven publication to democratize access and accelerate research, it has exposed the challenges of this model of scholarly communication. With intense public appetite for information about the virus and pandemic, there has been a real danger that individuals and institutions will act hastily on information about risks, mitigation strategies and treatments before it is adequately scrutinized. Such opportunities and challenges, best illustrated during the pandemic, lie ahead as medicine moves towards the goal of providing evidence-based care to patients no matter where they live. This trajectory also opens up real opportunities for speed, transparency and rich evaluation across peer review in medicine.

We are therefore excited to announce that eLife’s reinvigorated Medicine section will offer a whole new approach to publishing in medicine, including public health and health policy. We will apply our system of editorial oversight by practicing clinicians and clinician-investigators, and rigorous, consultative peer review to produce public reviews of preprints with significant potential to impact clinical practice. Our goal is to transform unrefereed manuscripts posted on medRxiv into refereed preprints that provide readers and potential users with a detailed assessment of the science, comments on its potential impact, and perspectives on its use. In essence, by providing rich and rapid evaluation of preprints, we hope that refereed preprints become a currency of trust in medicine.

We will continue to operate like a traditional journal, providing authors who submit their preprints to us with feedback from the reviewers and editors, and we will select a subset of papers for formal publication in eLife. Our scope is broad, covering all areas of the health sciences ranging from cellular and murine models of disease, to human genetics and genomic sciences, to therapeutic discovery, to all phases of clinical investigation, to population health outcomes, to health policy and clinical decision making.

eLife was launched in 2012 by three funders – Howard Hughes Medical Institute, Wellcome Trust and Max Planck Society, later joined by the Knut and Alice Wallenberg Foundation – who were eager to take a more active role in promoting best practices in scientific and medical publishing. Founding Editor-in-Chief Randy Schekman focused on improving the culture of peer review, pioneering a consultative approach in which reviewers and editors come together to discuss their assessments, reach a collective decision on whether the strengths of the work merit publication in eLife and which, if any, weaknesses need to be addressed before publication. The authors are then provided with a clear and concise decision letter that is free of the conflicting opinions and recommendations often found in reviews.

Since 2019, with one of us (MBE) as Editor-in-Chief, eLife has moved to embrace and encourage the growth of preprints even more strongly in all areas of science and medicine, and has developed a new concept of 'publish, then review' to serve readers and users, as well as authors (Eisen et al., 2020). This new system of creating public reviews of preprints, described briefly above, is the first major product of these efforts. The re-launch of eLife’s Medicine section is the second.

While eLife has traditionally focused on basic science, many of our editors are practicing clinicians who run research groups that cross the proverbial line between basic and clinical research. We have published papers in early translational research, but over the past few years have received many requests to bring our innovations in peer review and preprint review into the full spectrum of research in medicine.

To answer these calls, and to ensure we do so successfully, we have recruited two Deputy Editors in Medicine (DMH and MZ) and a group of Senior Editors that includes accomplished women and men from around the globe. These Senior Editors in turn have the responsibility of overseeing the review process through our Board of Reviewing Editors, which has also been greatly expanded (and will continue to expand) to include clinicians, public health specialists, and basic, translational and clinical researchers spanning a wide range of disciplines. In building the team, we have emphasized not only clinical expertise, scientific excellence and intellectual breadth, but also equity, diversity and inclusion, in order to ensure that we address patient-centered research for everyone.

The result is an editorial board that can truly aim to cover all areas of modern biology and medicine, with the ability to handle any paper in any discipline, especially those that span and knit together work from fields whose practitioners do not traditionally publish in or read the same journals. For example, we are now well equipped to evaluate all aspects of papers whose topics range from behavioral sciences and social determinants of health to clinical genetics, structural biology, drug discovery and early–stage efficacy studies. We are equally equipped to handle papers describing new COVID-19 virus mutations, their coverage by vaccines, and modeling future infections in different population settings.

In keeping with our desire to tackle the most difficult issues in publishing, we have also created a new Ethics Committee – a think tank of ethicists and individuals with long-standing experience in different aspects of science, medicine and publishing – to provide guidance on issues including, but not limited to, publishing, medical and animal ethics, biosecurity and biosafety, environmental justice, competing interests, data availability, and issues surrounding studies with minority populations in the developing world. These issues are of growing importance across both biology and medicine.

Over the past few years we have received many requests to bring our innovations in peer review and preprint review into the full spectrum of research in medicine.

We are also aware that the pipeline for the young physician-scientist is extremely leaky with most medical graduates who enter science being drawn into private medicine, whether or not they obtained a doctoral degree (Williams et al., 2018). We will leverage eLife’s expanded Medicine section to assist the careers of both laboratory-based physician-scientists and physicians whose interests lie in clinical investigation and health services research. In addition, we will encourage the STEM pipeline of women and underrepresented minorities in physician-scientist roles. eLife already has a program to encourage early-career researchers in all fields to become involved in the peer-review process, and we will also test novel processes for the mentorship, sponsorship and professional advancement of junior physician-scientists.

In all, eLife's ambitions in medicine are broader than just becoming a new open-access medical journal. This is a larger effort underscoring a cultural change to emphasize the importance of preprints and reviewing preprints; to focus on transparency, not just on open access but also on open data and open methods; and to encourage responsible behaviors in medical publishing – elements that are necessary for the translation of meaningful scientific investigation to the betterment of human health. Towards this aspiration, eLife’s reinvigorated Medicine section will cherish the support of the physician–scientist community around the globe.

## References

[bib1] Eisen MB, Akhmanova A, Behrens TE, Harper DM, Weigel D, Zaidi M (2020). Implementing a "publish, then review" model of publishing. eLife.

[bib2] Williams CS, Iness AN, Baron RM, Ajijola OA, Hu PJ, Vyas JM, Baiocchi R, Adami AJ, Lever JM, Klein PS, Demer L, Madaio M, Geraci M, Brass LF, Blanchard M, Salata R, Zaidi M (2018). Training the physician-scientist: views from program directors and aspiring young investigators. JCI Insight.

